# Unraveling the genomic underpinnings of unbalanced *MYC* break-apart FISH results using whole genome sequencing analysis

**DOI:** 10.1038/s41408-023-00967-8

**Published:** 2023-12-19

**Authors:** Marie-France Gagnon, Alan R. Penheiter, Faye Harris, Dorsay Sadeghian, Sarah H. Johnson, Giannoula Karagouga, Alexa McCune, Cinthya Zepeda-Mendoza, Patricia T. Greipp, Xinjie Xu, Rhett P. Ketterling, Ellen D. McPhail, Rebecca L. King, Jess F. Peterson, George Vasmatzis, Linda B. Baughn

**Affiliations:** 1 Division of Laboratory Genetics and Genomics, Department of Laboratory Medicine and Pathology, Mayo Clinic, Rochester, MN USA; 2 Center for Individualized Medicine, Mayo Clinic, Rochester, MN USA; 3 Division of Hematopathology, Department of Laboratory Medicine and Pathology, Mayo Clinic, Rochester, MN USA

**Keywords:** B-cell lymphoma, Cancer genomics

## Introduction

*MYC* rearrangements (*MYC*-R) constitute an integral defining feature in the diagnostic classification of mature aggressive B-cell lymphoma (BCL) [1–3]. Specifically, diffuse large B-cell lymphoma (DLBCL)/high-grade B-cell lymphoma (HGBCL) with *MYC* and *BCL2* rearrangements (and/or *BCL6* rearrangements) circumscribe a subset of higher-risk tumors. Current guidelines recommend investigating *MYC*-R with fluorescence in situ hybridization (FISH) [4]. In accordance with the diversity of rearrangement partners including immunoglobin (IG) and non-IG partners and the variability of breakpoints in the *MYC* locus, a break-apart (BAP) FISH probe is commonly utilized. Dual-color dual-fusion FISH probes (D-FISH) spanning *MYC* and IGH, IG-lambda (IGL) or IG-kappa (IGK) may also be used. We have previously demonstrated that in suspected HGBCL unbalanced rearrangements are identified in 11.9% of cases with abnormal results with the *MYC* BAP probe. In approximately 8.5% of cases, these cannot be reconciled with IGH/*MYC* D-FISH results and remain of ambiguous significance [5]. Forty-three percent of unbalanced cases also harbor a concurrent *BCL2* rearrangement (unpublished data), thereby emphasizing the importance of informed interpretation of results for accurate diagnostic classification and therapeutic management. In this study, we sought to elucidate the significance of these unbalanced rearrangements with whole genome sequencing (WGS) analysis.

## Materials and methods

### Survey

An online survey comprising 6 questions was distributed to the International Cytogenetic community via email (Supplementary Methods) to delineate the scope of FISH strategies used to investigate *MYC*-R and the interpretation practices of unbalanced *MYC* BAP results across clinical laboratories.

### FISH analysis

**Fig. 1:**
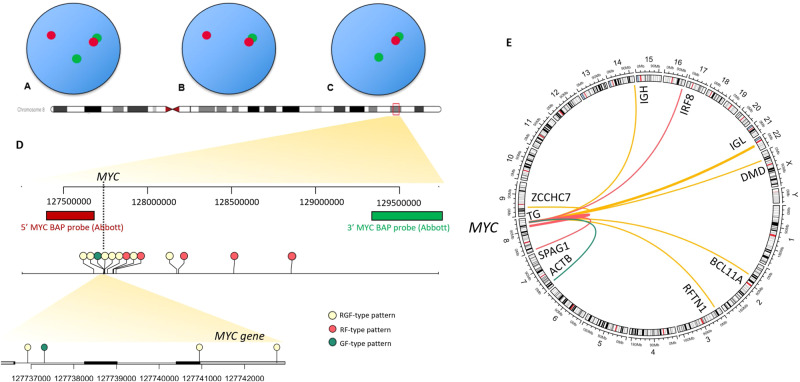
Representation of findings obtained with whole-genome sequencing and correlation with fluorescence in situ hybridization results. **A** Representation of a typical red (R)-green(G)-fusion(F)-type pattern, **B** Representation of an unbalanced RF-type pattern, **C** Representation of an unbalanced GF-type pattern, **D** Illustration of breakpoints identified at the *MYC* locus in our cohort with whole-genome sequencing (GRCh38 reference genome, created with Gviz and trackViewer R packages), **E** Circos plot illustrating *MYC* rearrangement partners identified in our cohort (created with circlize R package).

FISH analysis consisted of commercial *MYC* BAP and *MYC*/IGH D-FISH probe sets (Abbott Laboratories, Des Plaines, IL). The *MYC* BAP probe set included a red (R) and a green (G) probe which respectively hybridized 5′ and 3′ to the *MYC* gene, yielding a fusion (F) signal in the setting of an intact *MYC* locus. While typical *MYC*-R are indicated by balanced, separate red (R) and green (G) signals (RGF-type pattern), unbalanced patterns represent unbalanced or isolated R or isolated G signals. Cases with isolated R signals in the absence of isolated G signals (such as 1R1F) were referred to as RF-type patterns, and cases with isolated G signals in the absence of isolated R signals (such as 1G1F), were referred to as GF-type patterns (Fig. 1A).

### Whole genome sequencing

WGS was performed with DNA extracted from formalin-fixed, paraffin-embedded (FFPE) sections using Qiagen AllPrep or DNA FFPE kits (Cats #80234, #56404). A modified Covaris fragmentation protocol designed to capture larger insert sizes was used [6]. Libraries were multiplexed on an Illumina NovaSeq S4. Mapping to the GRCh38 reference genome and structural variant calling were performed with BIMA 3.1.5/SVAtools pipeline [6]. FFPEseq mean and range of uniquely mapped fragments for the 14 libraries was 492 M (386M-685M). Tumor bridge coverage (average number of fragments (read-pairs) spanning a position in the genome), adjusted for library insert length and pipeline estimated tumor percentage, was 30.5× (14.2×–61.4×).

### Clinical evolution

Baseline demographic characteristics, management approaches and response to treatment were extracted from medical chart review.

This study was approved by the Mayo Clinic Institutional Review Board (15-007359).

## Results

### Survey of interpretation practices of unbalanced *MYC* break-apart results

Fifty-four responses were obtained to the survey querying laboratory practices regarding FISH strategies and interpretation of unbalanced *MYC* BAP results. The survey participants were derived from ≥ 31 different institutions located in ≥4 different countries (23 responders did not provide information related to their work institution). Twenty-three of 54 laboratories (43%) only performed the *MYC* BAP probe and 30/54 (56%) laboratories performed the *MYC* BAP and IGH/*MYC* D-FISH probes upon initial investigation. One of 54 laboratories (2%) performed the *MYC* BAP, IGH/*MYC*, IGK/*MYC* and IGL/*MYC* D-FISH probe sets upon initial investigation. *BCL2* and *BCL6* rearrangements were sought upfront by 36/54 (67%) responders and 14/54 (26%) only queried these rearrangements in the event of a *MYC*-R. Thirty-six percent and 42%, respectively, reported interpreting RF- and GF-type patterns as a *MYC-R*, 58 and 56% reported RF- and GF-type patterns as equivocal, respectively, and 6 and 2% reported RF and GF-patterns as negative for a *MYC-R*, respectively (Supplementary Fig. 1). These data demonstrate significant variability in the interpretation of unbalanced *MYC* BAP results with most laboratories reporting an equivocal result.

### Cohort description and associated FISH analysis results

Our cohort included 14 cases of DLBCL/HGBCL evaluated in our clinical FISH laboratory between 2019 and 2021 and selected sequentially in inverse chronological order of sampling (with preference given to internal cases for clinical correlation). Seven cases had an unbalanced *MYC* BAP result, including 5 cases with a RF-type pattern and 2 cases with a GF-type pattern on *MYC* BAP analysis in the absence of an IGH partner by D-FISH. Seven specimens with a typical RGF-type pattern with a known *MYC*-IGH (*n* = 1) or unknown (*n* = 6) partner were also included. These served as controls to verify the ability of the WGS methodology from FFPE sections used in this study to identify rearrangements and dissect the genomic architecture at the *MYC* locus.

### Whole genome sequencing results

A *MYC-R w*as confirmed in all control cases with a balanced pattern on BAP FISH (Table 1, Fig. 1). These involved previously reported rearrangement partners/loci (IGH (*n* = 1), IGL (*n* = 2), *ZCCHC7* (*n* = 1), *RFTN1* (*n* = 1), *DMD* (*n* = 1) and *BCL11A* (*n* = 1)). A structural variant (SV) involving the *MYC* region was also detected in all 5 cases with a RF-type pattern. In line with the higher number of R signal(s) observed on BAP FISH, a relative gain of genomic material 5′ of the *MYC* gene or relative loss of material 3′ of the *MYC* gene was detected with WGS in all these cases. Putative fusion events juxtaposing *MYC* to (1) an intergenic region upstream of *TG*, (2) *TG*, (3) *IRF8*, and (4) *SPAG1* was detected in 4/5 cases, while case 5 involved a copy number (CN) gain of *MYC*. WGS also allowed to resolve the unbalanced FISH results for cases with a GF-type pattern and revealed SVs leading to a higher copy number (CN) of the 3′*MYC* BAP FISH probe-binding sequence in comparison with the 5′ region in both cases with a GF-type pattern, thereby also reconciling BAP FISH results. The first case with a GF-type pattern juxtaposed *MYC* with *ACTB* and the second involved a CN loss including *MYC* and the 5’ FISH probe-binding sequence. Of all cases with unbalanced FISH results, this case represented the only one in which WGS revealed a deletion involving *MYC* in our study cohort. *MYC* overexpression by immunohistochemistry ( ≥ 40%) was detected in 4/5 and 2/2 cases with RF- and GF-type patterns, respectively, suggesting that in 6/7 cases, the unbalanced *MYC* rearrangement was associated with increased MYC expression. In 7 cases with a typical pattern, overexpression of MYC was documented by IHC.

**Table 1:** Correlation of fluorescence in situ hybridization and whole-genome sequencing results at the *MYC* locus in 14 patients with DLBCL/HGBL.

Case	Group	FISH nomenclature	WGS result summary	Breakpoint relative to *MYC*	Breakpoint relative to partner gene	*MYC* overexpression by IHC
1	RF	nuc ish(5’MYCx3,3′MYCx2)(5’MYC con 3′MYCx2)[42/100]/(MYCx3)[30/100]	*MYC*:*:TG* (deletion within copy gain of chr8 leading to this juxtaposition event)	Intergenic, telomeric to *MYC*	Intergenic, centromeric to *TG* (break within *KCNQ3*)	Yes
2	RF	nuc ish(5′MYCx2,3′MYCx1)(5′MYC con 3′MYCx1)[100]	*MYC*::*TG*	Intergenic, telomeric to *MYC*, centromeric to *PVT1*	*TG*, intron 26 (NM_003235.5)	Yes
3	RF	nuc ish,(5′MYCx2 ~ 3,3′MYCx1-2)(5′MYC con 3′MYCx1-2)[97/100]	*MYC*:*:IRF8*	Intergenic, telomeric to *MYC* and *PVT1*	*IRF8*, intron 2 (NM_002163.4)	Yes
4	RF	nuc ish(5′MYCx2 ~ 3,3′MYCx1-2)(5′MYC con 3′MYCx1-2)[61/100]	*MYC*::*SPAG1* Templated insertion of *MYC* telomeric to *ADAM7* *2. MYC::ADAM28*	1. Intergenic, telomeric to *MYC* and *PVT1* 2. Intergenic, centromeric to *MYC*	1. *SPAG1*, intron 10, (NM_003114.5) 2. *ADAM28/ADAM7*	No
5	RF	nuc ish(5’MYCx2 ~ 5,3’MYCx1 ~ 2)(5’MYC con 3’MYCx1∼2)[100]	Relative gain of *MYC*, break in *PVT1*	Telomeric to *MYC*, *PVT1*, intron 1 (NR_003367.3)	NA	Yes
6	GF	nuc ish(5’MYCx1 ~ 3,3’MYCx2 ~ 4)(5’MYC con 3’MYCx1∼3)[80/100]	*MYC*::*ACTB* (fusion predicted)	Genic, *MYC* intron 1	*ACTB*, Intron 2 (NM_001101.5)	Yes
7	GF	nuc ish(5’MYCx1,3’MYCx2)(5’MYC con 3’MYCx1)[68/100]	Relative copy number loss including *MYC* in the setting of copy number gain of chr8q	NA	NA	Yes
8	RGF	nuc ish(5′MYCx2 ~ 4,3′MYCx2 ~ 4)(5′MYC sep 3′MYCx1)[90/100]	*MYC*::IGH (with trisomy 8)	Genic, *MYC* intron 1	Intergenic, centromeric to IGHE	Yes
9	RGF	nuc ish(5′MYC,3′MYC)x2∼4(5′MYC con 3′MYCx1 ~ 2)[93/100]	*MYC*::*RFTN1* fusion predicted (with trisomy 8)	Genic, *MYC* exon 3 (NM_002467))	Intron 2 of *RFTN1* (NM_015150)	Yes
10	RGF	nuc ish(5′MYCx2 ~ 5,3′MYCx2 ~ 5)(5′MYC con 3′MYCx1 ~ 2)[100]	1. *MYC*::*DMD* 2. *CASC8*::*PTCHD1* 3. *CASC8*::*DMD* 4. *MYC*::*DMD*	1. Genic, *MYC* 3′UTR 2. Upstream of *MYC*, *CASC8* intron 4 (NR_117100) 3. Upstream of *MYC*, *CASC8* intron 4 4. Intergenic, telomeric to *MYC*	1. *DMD*, intron 1, (NM_000109) 2. Intergenic, centromeric/upstream of *PTCHD1* (NM_173495) 3. *DMD*, intron 1 (NM_00109) 4. *DMD*, intron 1 (NM_00109)	Yes
11	RGF	nuc ish(MYCx2)(5′MYC sep 3′MYCx1)[96/100]	*MYC*::*ZCCHC7* fusion predicted	Intergenic, Breakpoint centromeric to *MYC*	Intron 2 of *ZCCHC7* (NM_032226)	Yes
12	RGF	nuc ish(MYCx2)(5′MYC sep 3′MYCx1)[92/100]	*MYC*::IGLL5 fusion (2 rearrangements)	1. Intergenic, telomeric to *MYC*, upstream of *PVT1* 2. Intergenic, telomeric to *MYC* and *PVT1*	1. IGLL5, intron 1 (NM_001178126) 2. IGLL5, intron 1	Yes
13	RGF	nuc ish(MYCx2)(5′MYC sep 3′MYCx1)[94/100]	*MYC*::IGL	Telomeric to *MYC*, *PVT1* intron 1 (NR_003367.3) and deletion of *PVT1* (complex event)	Intergenic, downstream of (telomeric) IGLL5	Yes
14	RGF	nuc ish(MYCx2)(5′MYC sep 3′MYCx1)[100]	*MYC*::*BCL11A*	1. Intergenic, telomeric/downstream of *MYC* and *PVT1*	*BCL11A* intron 1 (NM_022893.4)	Yes

*NA* not applicable, *RGF* red-green-fusion-type pattern, *RF* red-fusion-type pattern, *GF* green-fusion type pattern, *FISH* fluorescence in situ hybridization, *WGS* whole-genome sequencing

### Clinical correlation

Clinical information was available for 3 cases with a RF-type pattern (Supplementary Table 2). While one case exhibited a favorable response to MR-CHOP chemotherapy, the other two cases remained refractory to therapy (R-CHOP and R-CHOP followed by R-ICE, polatuzumab+bendamustine respectively) and expired from disease. In the first case with a GF-type pattern, a favorable response to R-CHOP chemotherapy was exhibited; nonetheless, the patient expired from sepsis and multi-organ failure. In the second case, no systemic therapy was provided to the patient.

## Discussion

The relevance of elucidating the implications of unbalanced *MYC* BAP results is underscored by the variability of interpretative practices across clinical laboratories highlighted by the survey we distributed to the cytogenetics community. Our study reveals the presence of true SV juxtaposing the *MYC* locus with a partner gene in most of these cases (4/5 with a RF-type pattern and ½ with a GF-type pattern). The remaining two cases (1/5 with a RF-type pattern and 1/2 with a GF-type pattern) also displayed a SV involving the *MYC* locus, yet these consisted of CN alterations and would not be considered as *MYC*-R per current DLBCL/HGBCL classification schemes [1–3]. In all cases, material 5′ of *MYC* for RF-type patterns and 3′ of *MYC* for GF-type patterns was present at a higher copy number state relative to 3′ and 5′ regions, thereby explaining the unbalanced FISH results.

*MYC*-R in DLBCL/HGBCL are thought to be acquired through aberrant activation-induced cytidine deaminase (*AICDA*)-mediated somatic hypermutation (SHM) and class-switch recombination (CSR) [7]. While *AICDA* mediates SHM and CSR at the IG loci, off-target mutagenic activity of this enzyme may occur in lymphoma-associated oncogenes such as *MYC* and result in oncogenic rearrangements. Breakpoints associated with *MYC* rearrangements exhibit significant variability within the *MYC* region. They may occur in a region designated as the “genic cluster” which encompasses a segment ~1.5 kb upstream of the transcription start site and the first exon and intron of *MYC* [8]. In our cohort, one case with a GF-type pattern exhibited a breakpoint within intron 1, a mechanism which has been suggested to result in aberrant *MYC* expression through dissociation of the natural promoter regions P1 and P2 and regulatory sites, resulting in transcription initiation from a cryptic promoter P3 in intron 3 [9]. Breakpoints located downstream of *MYC* may result in aberrant activation through the acquisition of *MYC* super-enhancers within a topologically-associated domain (TAD) comprising *MYC*, thus allowing for *MYC* activation through long-range loop interactions favored by a common CTCF binding site located 2 kb upstream of *MYC* [10]. Heterologous enhancers conferred by the rearrangement event may promote aberrant *MYC* expression in this setting [11]. In line with these considerations and in support of their potential for *MYC* activation, 4/5 RF-type cases involved breakpoints located downstream of *MYC*.

By study design, all cases with unbalanced FISH results and a confirmed *MYC*-R by WGS involved a non-IG partner gene. Consequently, the potential significance of these rearrangements must be taken into the context of the unclear prognostic relevance of non-IG *MYC*-R [12, 13]. An additional interpretative consideration of cases with unbalanced FISH results relates to the observation that these involved novel non-recurrent non-IG partner genes. Accordingly, in the absence of RNA expression data, whether these result in *MYC* overexpression remains difficult to ascertain, yet these were associated with *MYC* overexpression by IHC. Also, recently Collinge et al. [14] demonstrated that unbalanced patterns with loss of R or G signals were associated with mRNA expression levels comparable to HGBCL with *MYC* and *BCL2* rearrangements exhibiting balanced *MYC* FISH results. Similarly, several of the *BCL2* rearrangement in RF-type cases involved non-IG partners. In light of a paucity of data on atypical *BCL2* rearrangement partners, their clinical significance remains ambiguous. Our study is further limited by a limited sample size which precluded a refined analysis of potential differential implications of different unbalanced patterns by FISH. A recent study suggested that while patterns with 5′*MYC* gains may adversely impact prognosis, a loss of 3′*MYC* may not portend similar deleterious implications [15].

**Fig. 2:**
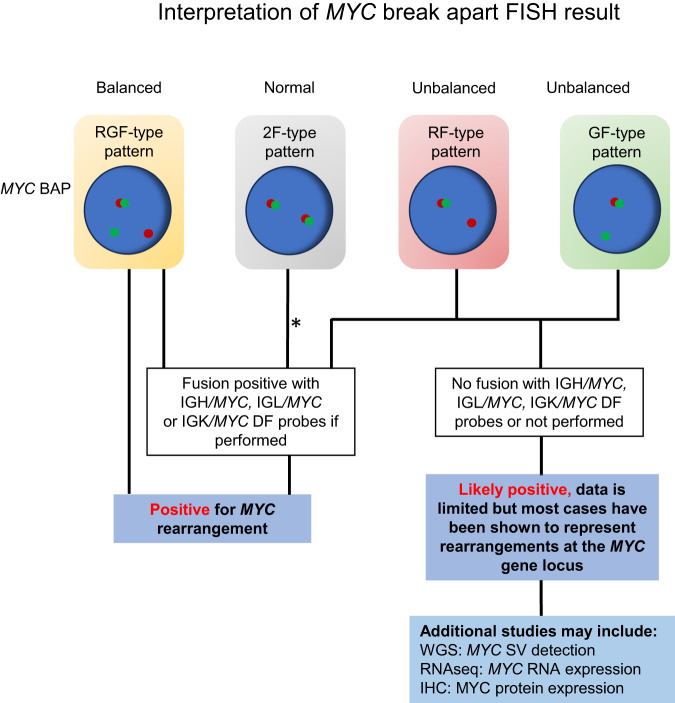
Proposed algorithm to guide the interpretation of unbalanced results obtained with the *MYC* break-apart (BAP) probe based on study results. Unbalanced patterns include RF (red-fusion)-type and GF (green-fusion)-type patterns. See text for further information. *It has previously been shown that evaluation for *MYC* rearrangements using only the *MYC* BAP is associated with a false negative (FN) rate of at least 4%. This FN rate can be reduced using IG/*MYC* D-FISH probes [16]. DF: probe dual color, dual-fusion probe set, IHC immunohistochemistry, RNAseq RNA sequencing, SV structural variant, WGS whole genome sequencing.

In all, our study reveals that true genomic SV involving the *MYC* locus often underlie unbalanced *MYC* BAP FISH results. While these SV may result in a juxtaposition of *MYC* with a rearrangement partner, less frequently, they may also be associated with a CN alteration at the *MYC* locus. Accordingly, our results counsel caution in the interpretation of the significance of these unbalanced BAP *MYC* signal patterns. While larger studies are needed to validate our findings, an interpretation rendered as ‘likely positive’ for a rearrangement at the *MYC* gene locus may be most judicious. While the significance of an unbalanced pattern by *MYC* BAP analysis cannot be ascertained with BAP FISH testing alone and data remains currently limited, most cases have been shown to represent true rearrangements at the *MYC* gene region (Fig. 2). This may allow communication of the atypical nature of results while also providing added context reflecting the current body of evidence for the provider. Additional testing may assist in further clarifying whether a true rearrangement juxtaposing *MYC* with a gene partner and resulting in *MYC* overexpression is present.

### Supplementary information

Supplemental material

## Data Availability

For original data, please contact Baughn.linda@mayo.edu.
