# Supplementary material for: Unraveling the genomic underpinnings of unbalanced MYC break-apart FISH results using whole genome sequencing analysis
Source: Blood Cancer J. 2023 Dec 19;13(1):190. doi: 10.1038/s41408-023-00967-8 (PMC10730864; doi:10.1038/s41408-023-00967-8)
Supplement: Supplementary file 1 — Supplemental material [file 41408_2023_967_MOESM1_ESM.docx]

**Supplemental methods**

FISH analysis

FISH analysis consisted of commercial *MYC* BAP and *MYC*::IGH D-FISH probe sets (Abbott Laboratories, Des Plaines, IL). A total of 100 interphase nuclei and 200 interphase nuclei were respectively analyzed per break-apart and dual color, dual fusion probe set by two qualified clinical cytogenetic technologists and interpreted by a board-certified (ABMGG) clinical cytogeneticist. The *MYC* BAP probe set included a red (R) and a green (G) probe which respectively hybridized 5′ and 3′ to the *MYC* gene, yielding a fusion (F) signal in the setting of an intact *MYC* locus. While typical *MYC*-R are indicated by balanced, separate red (R) and green (G) signals (RGF-type pattern) (**Figure 1A**), unbalanced patterns represent unbalanced or isolated R or isolated G signals. Thus, cases with isolated R signals in the absence of isolated G signals (such as 1R1F) (**Figure 1B**) were referred to as RF-type patterns, and cases with isolated G signals in the absence of isolated R signals (such as 1G1F) (**Figure 1C**), were referred to as GF-type patterns.

Whole genome sequencing analysis

WGS was performed with DNA extracted from formalin fixed, paraffin embedded (FFPE) sections using Qiagen AllPrep or DNA FFPE kits (Cats #80234, #56404). Extracted DNA was assessed for quantity with ThermoFisher Qubit fluorometer DNA kit and quality on an Agilent 2100 Bioanalyzer. The cut-off for continuation to library preparation was 15% of DNA fragments >1Kb and >100 ng DNA. New England Biolabs NEB Ultra II libraries were created following a modified Covaris fragmentation protocol designed to capture larger insert sizes which aid in structural variant calling^1^. Library QC was performed with Qubit HS DNA and NGS 7500 chip in the Agilent Bioanalyzer. Libraries were multiplexed on an Illumina NovaSeq S4. Mapping to the GRCh38 reference genome and structural variant calling were performed with BIMA 3.1.5/SVAtools pipeline^1^. FFPEseq mean and range of uniquely mapped fragments for the 14 libraries was 492M (386M-685M). Tumor bridge coverage, adjusted for library insert length and pipeline estimated tumor percentage, was 30.5X (14.2X – 61.4X).

Immunohistochemistry

Pathology reports were reviewed for immunohistochemistry (IHC) for *MYC* and *BCL2* expression. Results were available for all 14 cases.

**Survey questions and summary of survey results**

1. Which probe set combinations do you perform upfront to evaluate HGBCLs and DLBCLs?
   1. MYC break-apart probe only: n=23/54
   2. MYC break-apart and MYC/IGH dual color, dual fusion probes: n=30/54
   3. MYC break-apart and MYC/IGH, MYC/IGK, MYC/IGL dual color, dual fusion probes: n=1/54
2. What MYC BAP probe manufacturer do you use?

- Abbott Vysis : n=42/54
- Cytocell : n=5/54
- Metasystems: 4/54
- Other: 3/54

1. How do you interpret MYC BAP results with an unbalanced signal pattern (deletion of a 3’ signal) when utilizing your MYC BAP probe set without a known partner? (n=53 analyzable answers)
   - Positive for a MYC rearrangement: n=19/53
   - Negative for a MYC rearrangement: n=3/53
   - Equivocal: n=31/53
2. How do you interpret MYC BAP results with an unbalanced signal pattern (deletion of a 5’ signal) when utilizing your MYC BAP probe set without a known partner? (n=52 analyzable answers)
   - Positive for a MYC rearrangement : n=22/52
   - Negative for a MYC rearrangement: n=1/52
   - Equivocal: n=29/52
3. Approximately how many HGBCLs and DLBCLs do you evaluate per month using FISH for MYC?
   - 1-25: n=44/54
   - 26-50 n=7/54
   - 51-75: n=1/54
   - >75: n=1/54
   - Unknown: n=1/54
4. What is the name of your work institution? (This information will remain anonymous and confidential and will only be used for demographic purposes. Should you prefer not to answer this question, please proceed to the end of the survey.) (n=31 analyzable answers)
   - The survey participants were derived from ≥31 different institutions located in ≥4 different countries.

**Table S1.** Fluorescence in situ hybridization and whole-genome sequencing results for *BCL2* and BCL6 rearrangements

| Case | Diagnosis | *BCL2* | | | BCL2 overexpression by IHC | *BCL6* | | |
| --- | --- | --- | --- | --- | --- | --- | --- | --- |
|  |  | Rearrangement by FISH | Rearrangement by WGS | Rearrangement partner |  | Rearrangement by FISH | Rearrangement by WGS | Rearrangement partner |
| Unbalanced FISH findings – RF-type pattern | | | | | | | | |
| 1 | DLBCL, NOS | no | deletion of *BCL2* | *-* | yes | no | no | *-* |
| 2 | HGBCL with *MYC* and *BCL2* rearrangement | yes | yes | IGL | yes | yes | yes | IGH |
| 3 | HGBCL with *MYC* and *BCL2* rearrangement | yes | yes (4 junctions) | *CDR1* | yes | no | no | - |
| 4 | DLBCL, NOS | no | no | - | no | no | no | - |
| 5 | DLBCL, NOS | yes | yes | *CACNA1B, IL1R2* | yes | no | no | - |
| Unbalanced FISH findings –GF-type pattern | | | | | | | | |
| 6 | HGBCL with *MYC* and *BCL2* rearrangement | yes | yes | IGH | yes | no | no | - |
| 7 | DLBCL, NOS | no | no | - | yes | no | no | - |
| Balanced FISH findings – RGF-type pattern | | | | | | | | |
| 8 | HGBCL with *MYC* and *BCL2* rearrangement | yes | yes | IGH | yes | no | no | - |
| 9 | HGBCL with *MYC* and *BCL2* rearrangement | yes | yes | IGH | yes | no | no | - |
| 10 | HGBCL with *MYC* and *BCL2* rearrangement | yes | yes (+18q) | IGH | yes | no | no | - |
| 11 | HGBCL with *MYC* and *BCL2* rearrangement | yes | yes | IGH | yes | yes | no | - |
| 12 | HGBCL with *MYC* and *BCL2* rearrangement | yes | yes (+18) | IGH | yes | no | no | - |
| 13 | HGBCL with *MYC* and *BCL2* rearrangement | yes | yes (+18) | IGH | yes | yes | yes (inv3 or t(3;3)) | - |
| 14 | DLBCL, NOS | no | no | *-* | yes | no | no | - |

**Table S2.** Clinical evolution of cases included in study cohort

|  |  | Age at diagnosis | Management approach | Response to treatment |
| --- | --- | --- | --- | --- |
| Unbalanced FISH findings – RF-type pattern | |  |  |  |
| 1 | DLBCL, NOS | 60 | R-CHOP | Primary refractory disease, deceased (<1 month after diagnosis) |
| 2 | HGBCL with *MYC* and *BCL2* rearrangement | 85 | MR-CHOP | Complete response and ongoing remission (for 3 years) |
| 3 | HGBCL with *MYC* and *BCL2* rearrangement | 45 | Not available | Not available |
| 4 | DLBCL, NOS | 83 | Not available | Not available |
| 5 | DLBCL, NOS | 73 | R-CHOP followed by recurrence, treated with R-ICE, polatuzumab + bendamustine | Relapsed/refractory disease, deceased (5 months after diagnosis) |
| Unbalanced FISH findings –GF-type pattern | |  |  |  |
| 6 | HGBCL with *MYC* and *BCL2* rearrangement | 83 | R-CHOP | Favorable response but death from sepsis and multi-organ failure (6 months after diagnosis) |
| 7 | DLBCL, NOS | 64 | No systemic therapy | Death (<1 month after diagnosis) |
| Balanced FISH findings – RGF-type pattern | |  |  |  |
| 8 | HGBCL with *MYC* and *BCL2* rearrangement | 64 | EPOCH-R | Favorable response and ongoing remission (for 2 years) |
| 9 | HGBCL with *MYC* and *BCL2* rearrangement | 39 | R-CODOX-M/R-IVAC | Favorable response and ongoing remission (for 2 years) |
| 10 | HGBCL with *MYC* and *BCL2* rearrangement | 58 | R-CODOX-M x1 and DA-EPOCH-R x5 | Favorable response and ongoing remission (for 3 years) |
| 11 | HGBCL with *MYC* and *BCL2* rearrangement | 82 | Solumedrol | Decline in status, deceased (<1 month after diagnosis) |
| 12 | HGBCL with *MYC* and *BCL2* rearrangement | 80 | CAR-T cell therapy for relapsed transformed lymphoma | Complete response and remission for 1 year (subsequent death without recognized evidence of disease) |
| 13 | HGBCL with *MYC* and *BCL2* rearrangement | 73 | MR-CHOP followed by R-ICE for progression | Decline in status, deceased (<1 year after diagnosis) |
| 14 | DLBCL, NOS | 83 | Not available | Not available |

MR-CHOP: high-dose methotrexate and R-CHOP, R-CHOP: rituximab, cyclophosphamide, hydroxydaunorubicin, vincristine, prednisone, R-EPOCH: rituximab, etoposide, prednisone, vincristine, cyclophosphamide, hydroxydaunorubicin, R-CODOX-M/R-IVAC: rituximab, cyclophosphamide, vincristine, doxorubicin, high-dose methotrexate / rituximab, ifosfamide, etoposide, high-dose cytarabine

**Supplemental Figure 1.** Representation of survey results with respect to interpretation of unbalanced *MYC* break-apart fluorescence in situ hybridization (FISH) results. A) FISH strategies performed to interrogate *MYC* rearrangements B) Interpretation practices of survey responders for red-fusion (RF)-type patterns and for green-fusion (GF)-type patterns. BAP: break-apart, D-FISH: dual color, dual fusion. Created with ggplot2 R package.


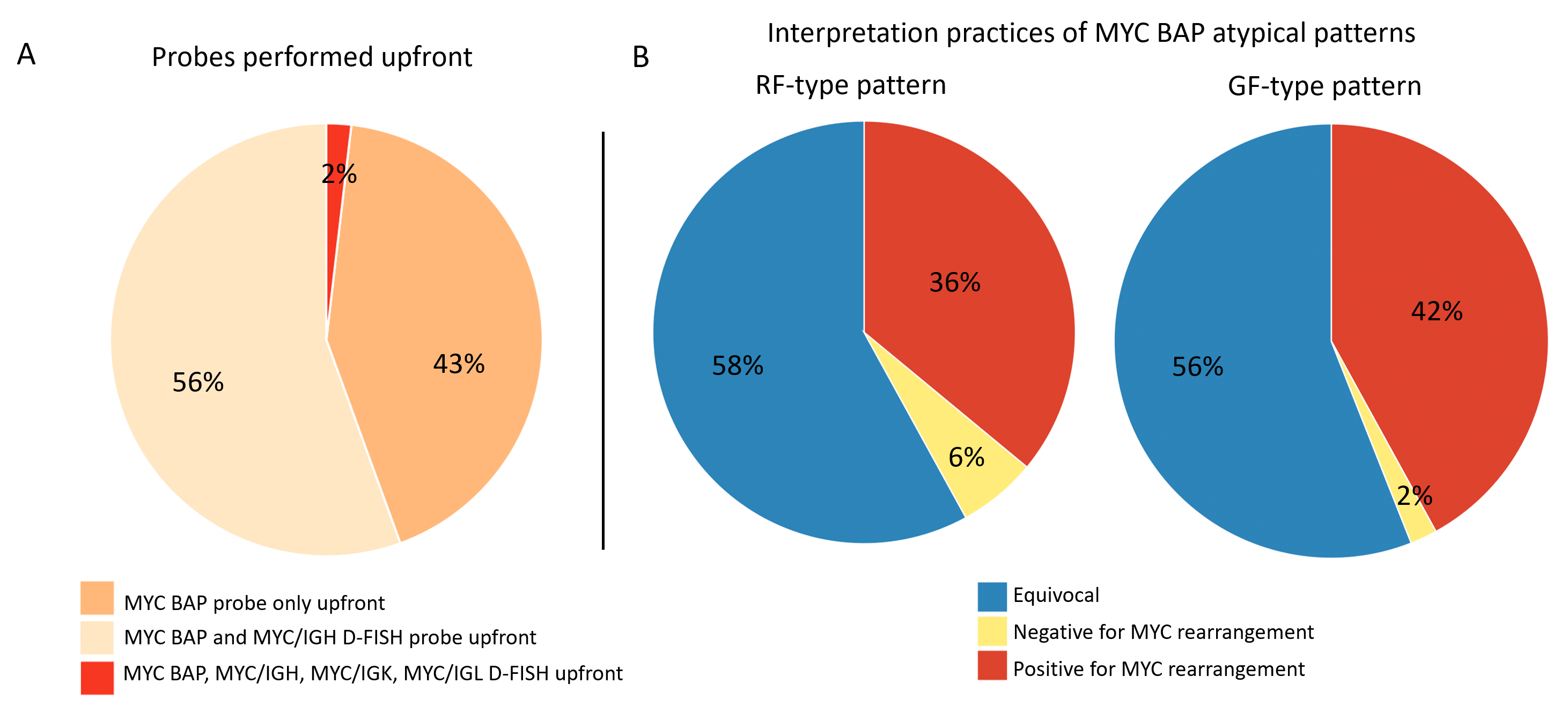


**References**

1. Murphy S, Smadbeck J, Eckloff B, et al. Chromosomal Junction Detection from Whole-Genome Sequencing on Formalin-Fixed, Paraffin-Embedded Tumors. *J Mol Diagn.* 2021;23(4):375-388.
